# Sleep Deficiency is a Modifiable Risk Factor for Obesity and Cognitive Impairment and Associated with Elevated Visfatin

**DOI:** 10.3889/oamjms.2015.063

**Published:** 2015-06-10

**Authors:** Yusr M. I. Kazem, Salwa M. El Shebini, Maha I. A. Moaty, Suzanne Fouad, Salwa T. Tapozada

**Affiliations:** *Nutrition and Food Sciences Department, National Research Centre, Dokki, Giza, Egypt (Affiliation ID: 60014618)*

**Keywords:** Sleep, cognitive functions, obesity, visfatin, insulin resistance

## Abstract

**AIM:**

To study the interaction between sleep deprivation, obesity and cognitive functions, and the effect of following a balanced low caloric diet and increasing sleep duration on those variables.

**SUBJECTS AND METHODS:**

Ninety two obese females with mean age 47.00 ± 2.00 years and body mass index (BMI) 36.14 ± 3.00 kg/m² were divided into 3 groups according to their sleeping hours. They followed balanced low-caloric diet and were instructed to increase sleeping hours. Full clinical examination, 24 hours dietary intake recall, anthropometric measurements, mini mental state test, questionnaire for subjective sleep and life style evaluation were performed at baseline and after 2 months. Serum visfatin, fasting blood glucose and C-peptide were assessed; Modified homeostatic model assessment of insulin resistance was calculated.

**RESULTS:**

About one third of our sample slept less than 6 hours daily, group (1), all patients had elevated visfatin serum level (33.87 ± 2.8 ng/ml) with the highest level in group (1). At base line, group (1) showed the highest BMI, lowest cognitive functions, highest visfatin level and highest insulin resistance (P < 0.05). After 2 months of intervention, improvement was recorded in all variables, with the best improvement in group (1) after extending sleep duration (P < 0.05).

**CONCLUSION:**

Sleep deprivation may be a modifiable risk factor for obesity, cognitive impairment and visfatin elevation.

## Introduction

Obesity is a leading cause of morbidity and mortality in the 21^st^ century and its prevalence has been rapidly increasing over the past 30 years. Recently, a similar growth in self-reported sleep deprivation has been noticed, paralleling the obesity epidemic. It is estimated that average sleep time has fallen in the general population by an average of 2 hours per night [1, 2]. Chronic sleep deprivation and obesity may be two linked phenomena with similar epidemiology [3]. Chronic sleep deprivation has been associated with many chronic conditions. This includes increased body weight, glucose intolerance, cardiovascular disease, hypertension, cognitive impairment and impaired immune system function. Several epidemiological studies have shown that people who report sleeping less than 6.5 hours are at greater risk of gaining weight over time. Sleep may be an important regulator of energy metabolism in peripheral tissues [4, 5]. On the other hand, excessive sleep duration also has been associated with increased morbidity, suggesting a U-shaped curve between sleep duration and morbidity/mortality [6].

Sleep and the internal circadian clock influence a host of endocrine parameters, which alters multiple metabolic pathways, leading to insulin resistance, possibly decreased energy expenditure, increased appetite, and immunological changes [7]. It was reported that sleep deprivation predominantly interferes with attention and memory [8]. In turn, this was proved to affect cognitive function and increase the risk of Alzheimer. An interesting study reported that Low levels in the cerebrospinal fluid of β amyloid 42 (Aβ 42), which signifies the presence of amyloid plaques, are associated with poor sleep efficiency [9]. Poor sleep puts people at a greater risk for cognitive impairment. A study reported that getting even 1 hour less than the required sleep (7 to 8 hour nightly) leads to problems with memory, concentration and attention [10]. Furthermore, sleep extension can improve neurocognitive functions in chronically sleep deprived obese individuals [11]. Interestingly, six days of partial sleep restriction increased the activation of food reward pathways –a putative pathway leading to weight gain [12].

Low-grade systemic inflammation due to obesity is considered to be the key link between obesity and obesity-related disorders which include cognitive impairment [1]. Nicotinamide phosphoribosyltransferase (Visfatin) is an adipocytokine produced primarily by visceral adipose tissue. Visfatin exerts insulin-mimetic effects via binding and activating the insulin receptor. In addition to its effect on the insulin receptor, it is a proinflammmatory cytokine with accumulating evidence for its rise in circulation, accompanying systemic inflammation [13]. Higher levels of visfatin were reported among obese in several studies. It has been proposed as playing a role in the pathogenesis of insulin resistance which is one of the causes of cognitive impairment. This was accompanied with higher levels of homeostatic model assessment of insulin resistance (HOMA-IR). In addition, several studies reported that short sleep time was predictive of higher Visfatin levels [14, 15].

The aim of this study was to test the effect of increasing sleeping hours and improving sleep quality on obesity and cognitive functions, as well as to examine serum visfatin in relation to the other variables under study, and to test the possibility of being used as a biochemical marker.

## Subjects and Methods

### Subjects

Ninety two obese females were included in this study with mean age of 47.00 ± 2.00 years and body mass index (BMI) of 36.14 ± 3.00 kg/m². They were all enrolled in a program for losing weight at the Nutrition Department, National Research Centre.

### Intervention lasted for 2 months

1 - All the participants were put on a balanced low-caloric diet (1000- 1200 K calories/day).
2 - After the first visit evaluation, the participants were divided into 3 main groups according to their sleeping hours per night, those who slept from 4-6 hours per night were instructed to make some changes in their life style in order to increase sleeping hours by 1-2 hours per night.

The participants were informed about the purpose of the study and their permission in the form of written consent was obtained. The protocol was approved by the “Ethical Committee” of the “National Research Centre”.

### Methods

All subjects were examined at baseline and after two months of following the balanced low-caloric diet (1000- 1200 K calories/day).

#### 1 - Full clinical examination

#### 2 - Mini Mental State Examination (MMSE)

was performed for evaluation of mental and cognitive status. The Mini Mental State Examination (MMSE) is the most commonly used test for complaints of memory problems [16].

It is a sensitive, valid and reliable 30-point questionnaire that is used extensively in clinical and research settings to measure cognitive impairment. It is also used to estimate the severity and progression of cognitive impairment and to follow the course of cognitive changes in an individual over time; thus making it an effective way to document an individual’s response to treatment. Administration of the test takes between 5–10 minutes and examines functions including registration, attention, calculation, recall, language, commands and orientation [17].

#### 3 - Sleep and life style questionnaire

A questionnaire designed to evaluate subjective sleep quality and number of sleeping hours and their pattern were evaluated. Evaluation of life style was achieved through questioning: Exposure to sun: time, duration and clothing. General subjective life stresses, mood, tea and coffee consumption, general activity and history of exercising, which were recorded and put on a 3 points scale.

Classification and scoring system: Sleeping hours: 4-6 =1, 6-8 =2 and 8-10 = 3; MMSE: 22-26 =1, 26-28=2 and 28- 29=3; Mood: bad = 1, regular =2 and good =3; Tea and coffee consumption (number of cups per day): 0-1 =1, 2-4 =2 and 5-8 =3. Exposure to sun, sleep quality, stress, general activity and exercising: 1 = low, 2 = medium, and 3 = high.

After the first visit evaluation, the patients were classified into 3 groups according to their total sleeping hours, group (1) consists of 29 patients who slept (4-6) hours per night, group (2) consists of 60 patients, they slept from (6-8) hours per night, and group (3) consists of 3 patients, who slept from (8-10) hours per night. Group (1) was instructed to make some changes in their life style in order to increase sleeping hours by 1-2 hours per night.

#### 4 - Anthropometric parameters and blood pressure measurements

Measurements of height and body weight were taken to calculate body mass index (BMI), where BMI= weight in kg/square height in meters. Blood pressure for each patient was measured weekly. All measurements were taken by the same researcher to assure accuracy.

#### 5 - Dietary recall.

Data on dietary intake before the intervention were performed using the 24 hours dietary intake recall, aiming to correct eating habits. All food items and portions were recorded by the same researcher in details.

#### 6 - Blood sampling and biochemical analysis.

Fasting blood samples (8 hours fasting) were drawn from the patients. Fasting blood glucose (FBG) was determined on fresh samples; other biochemical parameters were performed on the fasting blood sera that were stored at -70°C until used. The FBG was determined by using glucose oxidase method [18]. C-peptide was detected by C-peptide Enzyme Immunoassay Test Kit, catalogue No. E29-071, IMMUNOSPEC Corporation, Netherland, [19]. Modified homeostatic model assessment of insulin resistance (M. HOMA-IR) was calculated, where M. HOMA-IR = 1.5 + fasting blood glucose x fasting C-peptide/2800 [20], in which insulin was replaced by C-peptide so as to be applied on diabetic patients using exogenous insulin. Serum nicotinamide phosphoribosyltransferase (Visfatin) was measured by an enzyme-linked immunosorbent assay performed according to the directions of the supplier (*EIAab*^®^ Immunoassay Kit, Catalog No: E0638h) [21].

### Study design and statistics

This is an interventional study. Statistical analysis was performed using SPSS (17.0, 2008) software. Data are expressed as means ± SD and %. *t*–test for significance was used to compare the data. P -value was considered significant at < 0.05.

## Results

As shown in Table 1, base line results regarding sleep duration was 31.7% of the participants (29 subjects) which represent group (1) had very low hours of sleep (4-6 total sleeping hours per night), and 65 % (60 subjects) which represent group (2) get moderate sufficient hours of sleep (6-8 total sleeping hours per night), while 3.3% (3 subjects) represent group (3) get more than average hours of sleep (8-10 total sleeping hours per night). As for the mini mental state test which evaluate cognitive functions 10.7% of the participants have mild cognitive impairment, 80.5% have good mental functions and no signs of cognitive impairment, while 8.8% have excellent mental and cognitive functions. Sleep quality is low in 38.3% moderate in 36.9% and high in 24.8%. Criteria for sleep quality is subjective according to the patients recalling that they get up many times during night sleep and/or experiencing very shallow sleep that when they wake up they feel as if they were awake all night, also recalling feeling bad and exhausted, sometimes very sleepy, these are common comments from the patients suffering from low quality sleep. Exposure to sun is low in 48.5%, moderate in 31.3 % and high in 20.2%. Stress perception is recalled in 15.3 % as low, 49% as medium and 35.7% as high. General activity is low in 23%, medium in 55% and high in 22%. Exercising is recorded low in 41.8%, medium in 50.1% and high in 8.1 %. Bad mood is recorded in 26.4%, regular mood in 55.6 % and 18 % recorded good mood. The consumption of tea and coffee was low in 14.3 %, medium in 79.5 % and high in 6.2%.

**Table 1:** Data presented as percentage of obese women at base line stage, and after 2 months of intervention according to their sleeping hours

Variables	Score (1) % (no.)		Score (2) % (no.)		Score (3) % (no.)	

	Base line	After 2 months	Base line	After 2 months	Base line	After 2 months
Sleeping hours	31.7(29)	20 (18)	65 (60)	77 (71)	3.3 (3)	3 (3)
MMSE	10.7	4.3	80.5	86.7	8.8	9
Sleep Quality	38.3	31.8	36.9	43.2	24.8	25
Exposure to sun	48.5	47	31.3	33	20.2	20
Stress	15.3	17	49	48	35.7	35
General Activity	23	11.5	55	65.5	22	23
Exercising	41.8	36.4	50.1	55.6	8.1	8
Mood	26.4	18.6	55.6	62	18	19.4
Tea and coffee consumption	14.3	24	79.5	71	6.2	5

MMSE: Mini Mental State Examination; Sleeping hours: 4-6 = 1, 6-8 = 2, 8-10 = 3; MMSE: 22-26 = 1, 26-28 = 2, 28- 29 = 3; Mood: bad = 1, regular = 2, good = 3; Tea and coffee consumption (number of cups per day): 0-1 = 1, 2-4 = 2, 5-8 = 3; Exposure to sun, sleep quality, stress, general activity and exercising: 1 = low, 2 = medium, 3 =high.

All participants showed marked improvement in sleep duration and quality, cognitive functions, general activity, exercising and mood after intervention. A trend towards reducing tea and coffee consumption is seen as a result of the instructions the participants received to help improving sleep.

Table 2 represents the data of the 3 groups classified according to sleep duration, where group (1) represents the least sleeping hours at base line (5.4 hours per night). They showed the lowest scores in the MMSE indicating lower cognitive functions and highest mean BMI, the highest level of visfatin serum level, the highest value of M.HOMA-IR indicating more insulin resistance. After 2 months of intervention, significant improvement was seen in cognitive functions, BMI, visfatin serum level and insulin resistance (P< 0.05). The other 2 groups also showed improved cognitive functions, reduced BMI and visfatin serum level and insulin resistance but to a lesser extent when compared to group (1).

**Table 2:** Data of different groups before and after intervention classified according to their sleeping hours /night and expressed as mean ± SD

Variables	Group (1) (No.=29)		Group (2) (No.=60)		Group (3) (No.=3)	
	Base line	After 2 months	Base line	After 2 months	Base line	After 2 months
Sleeping hours	5.4 ± 0.71	6.5 ± 0.94*	7.45 ± 1.2	7.89 ± 1.1	8.8 ± 1.4	8.9 ± 1.37
Visfatin (ng/ml)	34.8 ± 1.3	32.5 ± 1.7*	33.29 ± 1.9	31.2 ± 1.6*	33.19 ± 1.2	31.45 ± 1.1*
M. HOMA-IR	1.72 ± 0.031	1.63 ± 0.024*	1.68 ± 0.047	1.60 ± 0.03*	1.69 ± 0.03	1.61 ± 0.029*
MMSE	24.2 ± 2.0	25.6 ± 1.9*	27.1 ± 0.05	27.9 ± 0.03	28.5 ± 0.03	29.0 ± 0.021
BMI (kg/m²)	37.23 ± 1.8	33.2 ± 1.7*	35.8 ± 2.9	31.95 ± 2.8*	36.1 ± 2.3	31.56 ± 2.1*

M. HOMA-IR: Modified Homeostatic Model Assessment of Insulin Resistance, MMSE: Mini Mental State Examination, BMI: Body Mass Index.

* P -value is considered significant at< 0.05.

Table 3 shows significant reduction in BMI among all participants, accompanied by significant reduction in serum visfatin level, insulin resistance, fasting blood glucose and C-peptide. Our study shows elevated visfatin level among all groups at baseline. After the intervention significant reduction was recorded in the visfatin level but was still much higher than normal level.

**Table 3:** Data for all participants expressed as mean ± SD at the base line and after 2 months of intervention

Variables	Base line	After 2 months
BMI (kg/m²)	36.14 ± 3.0	32.8 ± 2.7*
FBG (mg/dl)	111.38 ± 4.0	98.45 ± 3.0*
C-peptide (ng/ml)	4.67 ± 0.98	3.69 ± 0.86*
M.HOMA-IR	1.69 ± 0.04	1.60 ± 0.03*
Visfatin (ng/ml)	33.87 ± 2.8	31.76 ± 2.5*

BMI: Body Mass Index, FBG: Fasting Blood Glucose, M. HOMA-IR: Modified Homeostatic Model Assessment of Insulin Resistance.

* P -value is considered significant at< 0.05.

## Discussion

Most studies put 6 hours of sleep as a cut off point for sleep deficiency, which if not achieved, several negative consequences follow.

In our study more than 30 % of the participants reported to sleep from 4-6 hours per day, group (1), which means that they are sleeping deficient. This group when compared to the other groups with more sleeping hours showed the highest BMI, lowest cognitive functions, highest levels of visfatin, and highest insulin resistance. After 2 months of intervention through following a balanced low caloric diet and increasing sleep duration in those sleep deficient, improvement was recorded in BMI, cognitive functions, insulin resistance and visfatin serum level, but the highest improvement level was recorded in group (1) as seen in Table 2. In our study, all participants are obese which puts the body in a low-grade inflammatory state, in group one with sleep deficiency, an additional risk factor is added, that is sleep deficiency. That’s why group (1) showed worse scores at base line and achieved the highest level of improvement after intervention. We suggest that sleep deficiency can be an additional risk factor for obesity, cognitive impairment, insulin resistance and low-grade inflammatory state.

### Sleep and obesity

In agreement with the data detected in this study, accumulating evidence has pointed to an association between sleep inadequacy and obesity. In the last 50 years, self-reported sleep duration in the United States has decreased by 1.5–2 hours due to lifestyle changes. According to the latest surveys, 36% of the adult population is obese and one third of adults report sleeping less than 6 hours per night, substantially less than the recommended 7–9 hours sleep per night [22]. Chronic sleep deprivation and obesity may be related in a bidirectional fashion, and they have similar consequences, including hypertension, diabetes, and cognitive impairment. Previous studies reported the gradual decline in the amount of time spent asleep and also the routine extension of normal activity during the night may disrupt synchrony between the periods of sleep/activity with alternating periods of feeding/fasting and energy storage/utilization. Indeed, the relationship between sleep restrictions, weight gain may involve at least in part alterations in glucose metabolism, stimulation of appetite, and decreased energy expenditure [23, 24].

Several epidemiological studies have shown that people who report sleeping less than 6.5 hours are at greater risk of gaining weight over time and reduced ability to lose weight. Sleep restriction increases food intake beyond the energetic costs of increased time spent awake due to disrupted appetite-regulating hormones, altered brain mechanisms involved in the hedonic aspects of appetite, and/or changes in sleep quality and architecture [3, 25].

It was reported that acute sleep deprivation can induce insulin resistance. There are several postulated mechanisms for the effect of sleep curtailment on development of insulin resistance as well as for predisposition for Type 2 diabetes: increase of sympathetic neuronal activity, decreased cerebral utilization of glucose, increase in evening cortisol values, growth hormone increase and disorder of neuroendocrine control of appetite which increases the risk for getting the body weight [26].

A meta-analysis of 45 cross-sectional studies, including 604,509 adults and 30,002 children, confirmed the relationship between short sleep and obesity, most of the included studies used actigraphy or self-reported sleep duration, women, but not men, who slept fewer than five hours, had a higher risk of gaining 5 kg in two years. Interestingly, in this study, BMI was also increased in women sleeping more than eight hours [27]. A study suggested the bidirectional effect of sleep-obesity suggesting that weight loss may improve sleep, and these improvements may promote further weight loss. Overall, improving sleep duration and quality is a potential tool to counteract the epidemics of obesity [3, 28].

### Sleep and cognition

In our study, group (1) with the least sleeping hours showed the least scores in the cognitive test evaluation linking sleep deficiency to cognitive impairment. Several studies support this link as they reported that obesity and sleep deprivation have been linked to cognitive deficits. Sleep deprivation decreases attention and impairs processing speed. Sleep quality was worst in participants with memory, attention and motor domains impairments. Memory function was correlated with sleep efficiency [29, 30]. Furthermore, sleep extension can improve declarative memory in adolescents. A study reported that extending sleep hours resulted in attention improved by 10%; memory and executive functions tended to improve by 7% and 5%, respectively [11]. A study reported that getting even 1 hour less than the required sleep (7 to 8 hours nightly) leads to problems with memory, concentration, and attention. A study reported that sleep extension in chronically sleep-deprived obese individuals who exhibited neurocognitive deficits was partially reversible; self-reported sleep quality, duration and sleepiness, all improved to a clinically meaningful extent as well. These improvements were achieved in a non-pharmacological way in a real life situation and were sustained over a long time. In conclusion, the findings suggest that night-time sleep is important for cognitive function [11]. Also some studies searched the link between sleep and Alzheimer’s disease (AD). Sleep is frequently impaired in individuals with AD. Sleep is probably one component of a variety of risk factors contributing to AD [31].

### Obesity and cognition

Several previous studies reported that obese individuals exhibit deficits in memory, attention, executive functions, including mental flexibility, planning, problem solving, and display impulsivity. Insulin resistance in obese patients and the low-grade inflammatory status play an important role in cognitive impairment [32]. The link between obesity and dementia in women was stronger than that in men. Women who developed dementia had a higher average BMI compared to women without dementia [33], this matches our results.

### Sleep and insulin resistance

In our study, insulin resistance was worst in sleep deficient group and showed the best improvement when sleeping hours were extended. Several cross-sectional studies showed that short sleep may now qualify as an additional clinical factor in the development of insulin resistance and diabetes. One possibility is sleep may impact secretion of adipocyte derived hormones that regulate inflammation and insulin resistance. As indicated by studies of sleep deprivation, the mechanisms connecting short sleep and obesity/insulin resistance are probably mediated by three pathways: increases in appetite, decreases in energy expenditure, and influences on glucose metabolism [3, 6, 34].

### Sleep, obesity and inflammation

In the present study, visfatin was found to be elevated in all patients as seen in Table 3 (33.87 ± 2.8 ng/ml vs. 17.3 ng/ml which is the reference level), with its highest level in group (1) with sleep deficiency (5.4 ± 0.71 sleeping hours per night). This may be explained as sleep deficiency is an additional factor that increases the state of low-grade inflammation in the body, adding to the original inflammatory status due to obesity. That is why obese patients have elevated visfatin serum level, but the group with sleep deficiency showed even higher level of visfatin.

Visfatin is a recently discovered adipokine produced and secreted primarily by visceral adipose tissue (VAT). Visfatin correlated positively with insulin and HOMA-IR in obese subjects [35]. Recent studies reported that the important role of Visfatin in the development of obesity, diabetes mellitus, and metabolic syndrome continues to rise. A study reported a significantly higher visfatin level in the obese subjects compared to the control group (median Visfatin level of 39.6 vs.17.3 ng/ml, p = 0.0006) [36]. In a group of people with total sleep time (TST) of 6.2 hours, each hour reduction in TST was associated with 14% increase in visfatin levels. Another study reported an inverse association between sleep duration and levels of both leptin and visfatin. These findings suggest reduced sleep may have detrimental effects on adipose tissue function and these effects may help explain the systemic inflammation and insulin resistance associated with sleep deficiency [37-39].

Low-grade systemic inflammation due to obesity is considered to be the key link between obesity and obesity-related disorders. Weight loss in combination with increased physical activity, a negative energy balance, and diet adjustment was associated with lower inflammation [38]. In addition, adipokines play important roles in the regulation of appetite and satiety, fat distribution, insulin sensitivity and insulin secretion, energy expenditure, inflammation, blood pressure, hemostasis, and endothelial function [40, 41].

In our study significant decrease in Visfatin level accompanied by improvement in cognitive functions was seen after the 2 months of following low caloric diet and increasing sleeping hours. We can interpret these changes to the weight loss and improved sleeping that reduced the inflammatory status of the body. An important study reported that the inflammatory status that is caused by obesity in itself can be a factor that makes behavioral symptoms, including depressive symptoms, cognitive impairment and sleep problems [42].

In conclusion, we are shedding the light on a dangerous change in life style which is sleep inadequacy, where about one third of our sample slept around 5 hours per night. This study suggests that sleep deficiency is proposed as a modifiable risk factor for obesity and cognitive impairment mediated through insulin resistance and low–grade inflammatory status. Awareness and guidance are needed to improve life style including healthy diet and adequate sleep to help controlling obesity and cognitive impairment.
